# Med-SRNet: GAN-Based Medical Image Super-Resolution via High-Resolution Representation Learning

**DOI:** 10.1155/2022/1744969

**Published:** 2022-06-20

**Authors:** Lina Zhang, Haidong Dai, Yu Sang

**Affiliations:** ^1^Teaching Supervision Department, Zhejiang College of Security Technology, Wenzhou 325016, China; ^2^School of Electronic and Information Engineering, Liaoning Technical University, Huludao 125105, China

## Abstract

High-resolution (HR) medical imaging data provide more anatomical details of human body, which facilitates early-stage disease diagnosis. But it is challenging to get clear HR medical images because of the limiting factors, such as imaging systems, imaging environments, and human factors. This work presents a novel medical image super-resolution (SR) method via high-resolution representation learning based on generative adversarial network (GAN), namely, Med-SRNet. We use GAN as backbone of SR considering the advantages of GAN that can significantly reconstruct the visual quality of the images, and the high-frequency details of the images are more realistic in the image SR task. Furthermore, we employ the HR network (HRNet) in GAN generator to maintain the HR representations and repeatedly use multi-scale fusions to strengthen HR representations for facilitating SR. Moreover, we adopt deconvolution operations to recover high-quality HR representations from all the parallel lower resolution (LR) streams with the aim to yield richer aggregated features, instead of simple bilinear interpolation operations used in HRNetV2. When evaluated on a home-made medical image dataset and two public COVID-19 CT datasets, the proposed Med-SRNet outperforms other leading edge methods, which obtains higher peak signal to noise ratio (PSNR) values and structural similarity (SSIM) values, i.e., maximum improvement of 1.75 and minimum increase of 0.433 on the PSNR metric for “Brain” test sets under 8× and maximum improvement of 0.048 and minimum increase of 0.016 on the SSIM metric for “Lung” test sets under 8× compared with other methods.

## 1. Introduction

Low-resolution (LR) medical images present reduced important pathological details and compromise the diagnostic accuracy. High-resolution (HR) medical images provide vital detailed anatomical information for clinical application and quantitative image analysis. However, image quality is often affected by tremendous limitations. So, super-resolution (SR) is an extremely crucial technique for medical image processing [1, 2].

Recently, CNN-based SR methods [3–10], have achieved surprising performance. The networks are tending to be deeper and deeper from the ﬁrst SRCNN [3] to deeper VDSR [4], DRRN [5] and MemNet [11], etc. and then to the very deep RCAN [12]. In addition, the whole networks in other effective methods are constructed by simply connecting a series of identical feature extraction modules, e.g., RDN [13], IDN [14], MSRN [15], and SRFBN [16], which indicates that the capability of each block is important. The GAN model [17] provides a new idea for image generation and also provides a model basis for HR image generation. SRGAN [18] is the first work to introduce GAN model into SR reconstruction, which has obtained higher image visual quality and more realistic image high-frequency details. However, the extracted features are often insufficient due to the relatively simple design of SRGAN generation network, which affects the quality of reconstruction. Subsequently, some new SR methods based on GAN models and deep convolutional networks are proposed to improve the quality of image SR at different levels [19–24].

Unsurprisingly, deep learning intensively exploits multi-scale features and HR representations and has achieved impressive results on numerous vision tasks [15, 25–30]. HRNet [31] and its variant HRNetV2 [32] have superior performance. But they ignore the appropriate use of LR representations for providing contextual information for HR representations.

**Figure 1:**
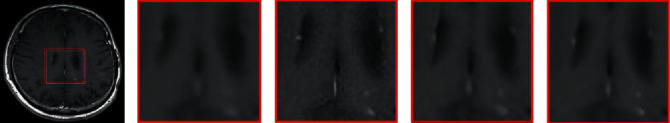
An example of medical image SR. (a) The original image. (b) The four red zones are the LR image (8×), (c) the original image (HR), (d) the SR image by DMSN (PSNR: 24.66) [25], and (e) the proposed Med-SRNet (PSNR: 25.59).

Although GAN-based SR models can achieve relatively satisfactory results, there are still some shortcomings: (i) the training process is unstable and the SR performance fluctuates greatly using original GAN framework; (ii) it is not suitable for extracting features in SR task because the generation network is too simple, resulting in insufficient image feature extraction and affecting the reconstruction quality. Therefore, we consider the advantages of GANs and CNNs to propose a novel GAN-based architecture for medical image SR via HR representation learning, namely, Med-SRNet. We modify the feature aggregation parts of HRNet and HRNetV2 and import HRNet framework to the SR task.  Figure 1 shows the SR result by Med-SRNet, indicating a clearer structure like the multiple punctate lesions in the red square regions. In summary, the contributions in this paper are threefold:

1. We use GAN as backbone of SR considering the advantages of GAN that can significantly reconstruct the visual quality of the images, and the high-frequency details of the images are more realistic in the image SR task.
2. We employ HRNet as backbone of SR to maintain the HR representations and repeat multi-scale fusions to strengthen HR representations for facilitating SR. Also, we adopt deconvolution operations to recover HR representations from the LR medical images with the aim to yield richer aggregated features, instead of simple bilinear interpolation operations used in HRNetV2.
3. We evaluate the proposed method with the constructed medical image dataset and two open-access COVID-19 CT datasets. The experimental results qualitatively and quantitatively demonstrated that the proposed method obtains higher PSNRs/SSIMs and preserves more local details and global features compared with other leading edge methods.

The rest of this paper is formed as follows. We present related work in Section 2.  Section 3 gives the proposed method. Performance evaluation is presented in Section 4. Conclusion with a brief summary is drawn in Section 5.

## 2. Related Work

In the last few years, signiﬁcant improvement of the SR quality has been achieved based on CNN models from the ﬁrst SRCNN [3] to the latest feedback network [16]. The superiority of the CNN-based SR methods over the conventional ones is remarkable. Due to the shallow structure, SRCNN shows poor performance. To boost the performance, the networks are getting deeper and deeper. For example, the VDSR model [4] proposed by Kim et al. has a deeper structure, and some recently proposed SR models with very deep structure, e.g., RCAN [12], achieve satisfying SR performance. Besides, dense connection-integrated SR models, e.g., SRDenseNet [6] and MemNet [11], further improve the performance. Moreover, some different forms of methods have been proposed [9, 10, 33]. Kong et al. [9] proposed the classSR framework to accelerate the SR network, and its classification method reduces the computational cost. Mei et al. [10] proposed a nonlocal sparse attention mechanism with dynamic sparse attention mode to achieve the robustness and efficiency of sparse representation while maintaining the ability of nonlocal remote modeling. Lin et al. [33] proposed an improved RCAN model, adding training iterations in the model training stage to improve the performance of the model. For representative computer vision tasks, i.e., object detection, image classification, and semantic segmentation, multi-scale networks [8, 15, 25–30] achieved outstanding results. For SR tasks, multi-scale networks [8, 15, 25] also have superior performance. A multi-scale residual network for image SR with the ability of adaptively detecting the image features at different scales was presented by Li et al. [15]. A multi-scale information distillation network for single image SR by Sang et al. [8] fully exploits image features and restores the LR images to HR ones with high efficiency. More relevant to this work, a deep multi-scale network (DMSN) for medical image SR by Wang et al. [25] enables a better representation of global topological structure and local texture detail of HR medical images. But the common deficiency of these multi-scale networks is high computational load caused by huge parameter number. To solve this problem, Sun et al. [31] proposed a building block by establishing hierarchical residual-like connections within one single residual block, called Res2Net, which is superior to the leading edge baseline methods. For better performance, Sun et al. proposed HRNet [31] and its variant HRNetV2 [32], which maintains HR representations through the whole process. However, HRNet and HRNetV2 ignore the appropriate use of LR representations for providing contextual information for HR representations. Besides, Guo et al. [34] proposed a deep wavelet SR (DWSR) network to recover the HR image from the LR image by predicting “missing details” of wavelet coefficients. Huang et al. [35] used wavelet transform in the CNN-based face SR for validation and they captured the accurate global topology information and local textural details of faces.

GAN-based SR methods have developed recently. SRGAN [18] is the first work to introduce GAN model into SR reconstruction, which has obtained higher image visual quality and more realistic image high-frequency details. Subsequently, some other GAN-based SR methods have proposed, including enhanced super-resolution generative adversarial network (ESRGAN) [19], deep convolutional generative adversarial network (DCGAN) [20], WGAN [21], patch GAN [22], conditional generative adversarial network (CGAN) [23], and so on. Wang et al. [19] proposed ESRGAN, which replaces the residual block with the dense block and removes the batch norm (BN) layer. Although the PSNR of the generated image is not ideal, the sensory effect is greatly improved. The discriminator of patch GAN [20] reduces the training parameters and makes the model lightweight and easy to train. Gao et al. [23] proposed CGAN-based image SR network. The possible mismatch between input and output when GAN is directly applied to SR is addressed, and its generator adopts a symmetric encoder-decoder structure and applies a skip connection to achieve cross-layer transfer of low-level information. Zun et al. [24] proposed a multi-scale parallel learning generative network structure based on SRGAN, which consists of two blocks of residual networks, learning the extracted LR images by the multi-scale characteristics of the two subnetworks and then using the fusion network to fuse the high-frequency information at different scales to generate HR images.

## 3. Proposed Method

In this section, we present the architecture of the proposed Med-SRNet. This work aims to reconstruct an SR medical image from an LR one, which is obtained by the bicubic operation of HR. Let *X* and *Y* denote the LR and HR images, respectively. The end-to-end mapping function *G*_*θ*_(·) between *X* and *Y* can be derived by solving the following problem:

(1) $$\begin{array}{c} \hat{\theta }=\text{arg}\underset{\theta }{\text{min}}\frac{1}{N}\sum_{i=1}^{N}L\left(G_{\theta }\left(X_{i}\right),Y_{i}\right), \end{array}$$

where *θ* is the network parameter set that needs to be optimized; *L*(.) is the loss function for minimizing the difference between *X* and *Y*; and *N* is the training sample number.

**Figure 2:**
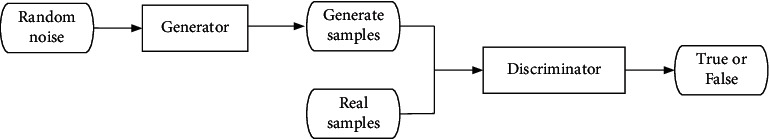
The network structure of GAN.

GAN [17] can be recognized as an effective framework. As shown in Figure 2, GAN is a generative model with zero-sum game thinking, consisting of a generator *G* and a discriminator *D*. The generator *G* falsifies the data by the initial input noisy data, while the discriminator *D* determines whether the input data are falsified by the generator or are the real data. The two play against each other repeatedly through such a process, which keeps sending back information and optimizing their network capabilities, respectively, until finally the discriminator *D* can accurately determine the authenticity of data while the generator *G* generates data powerful enough to blur the judgment of *D*.

Thus, following SRGAN [18], we further define a discriminator network *D*_*θ*_*D*__ in which we optimize in an alternating manner along with *G*_*θ*_*G*__ to solve the adversarial min-max problem:

(2) $$\begin{array}{c} \underset{\theta_{G}}{\text{min}}\underset{\theta_{D}}{\text{max}}E_{Y∼P_{\text{data}}\left(Y\right)}\left[\log D_{\theta_{D}}\left(Y\right)\right]+E_{X∼P_{\text{data}}\left(X\right)}\left[\log\left(1-D_{\theta_{D}}\left(G_{\theta_{G}}\left(X\right)\right)\right)\right], \end{array}$$

where *P*_data_(*X*) denotes the true sample distribution and *P*_data_(*Y*) denotes the generator distribution.

**Figure 3:**
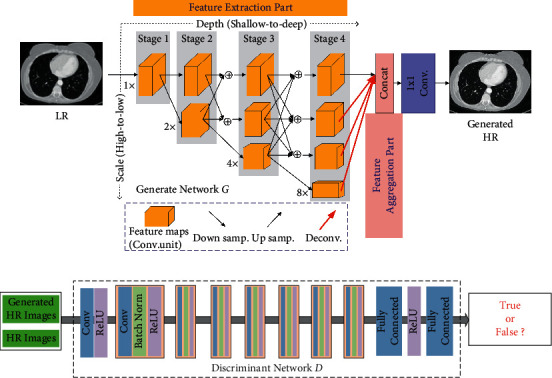
The architecture of the proposed Med-SRNet. (a) Generate network. (b) Discriminant network.

Figure 3 shows the complete architecture of the proposed Med-SRNet. It starts from LR images. Then, we use the HRNet backbone network to learn. Here, we mainly focus on the backbone network as shown in the feature extraction part and feature aggregation part of generate network in Figure 3.

For generate network as shown in Figure 3(a), we employ HRNet [31] as backbone of SR, which repeats use multiscale fusions to maintain HR representations through the whole process. However, it only uses the representation output from the highest resolution without feature aggregation. In its variant HRNetV2 [32], Sun et al. [31] aggregated the upsampled representations from all the parallel convolutions rather than only the HR representations. Inspired by Xiao et al. [36], deconvolutional layers can recover high-quality HR representations. So, we adopt deconvolution operation to recover HR representations from all the parallel LR images with the aim to yield richer aggregated features, as shown by the red up arrows in the feature aggregation part of Figure 3(a), instead of bilinear interpolation operation used in HRNetV2. It takes further experiment to demonstrate its effectiveness in Section 4.4.

For the feature extraction part, it starts from a HR subnetwork as the first stage and gradually adds high-to-low resolution subnetworks one by one to form more stages. Meanwhile, it connects the multi-resolution subnetworks in parallel. Multi-scale fusions are conducted repeatedly such that each of the high-to-low resolution representations receives information from other parallel representations over and over, leading to rich HR representations.

In the generator of Med-SRNet, we use 4 stages with 4 parallel subnetworks, similar to HRNet-W32 [31]. The resolution is smoothly halved while the channel number is doubled accordingly. The first stage is composed of 4 residual units, and each of them contains a 64-channel (width) bottleneck, and then the width will be reduced to 32 via a 3 × 3 convolution. Stages 2 to 4 contain 1, 4, and 3 convolution units, respectively. Every convolution unit has 4 residual blocks, each of which has two 3 × 3 convolutional layers. Then, we obtain 4 different widths (32, 64, 128, and 256). After that, we adopt 5 × 5, 7 × 7, and 11 × 11 deconvolutional layers on 3 lower resolution representations, respectively. Finally, four groups of HR representations are aggregated via concatenation operation, followed by one 1 × 1 convolution for prediction. All convolutional layers are followed by ReLU [37].

To discriminate real HR images from generated SR samples, we train a discriminator network, the same as SRGAN [18]. The general architecture is illustrated in Figure 3(b). Here we follow the architectural guidelines summarized by Radford et al. [20] and use ReLU activation, which avoids max-pooling throughout the network. The discriminator network contains eight convolutional layers with an increasing number (64 to 512) of filter kernels [38]. Strided convolutions are used to reduce the image resolution, and the number of features is doubled. The resulting 512 feature maps are followed by two dense layers and the sigmoid activation function to obtain a probability for sample classification.

Following SRGAN [18], the total loss function *L*_Total_^SR^ of the proposed model is defined as weighted sum of individual loss functions:

(3) $$\begin{array}{c} L_{\text{Total}}^{\text{SR}}=a_{1}L_{\text{MSE}}^{\text{SR}}+a_{2}L_{\text{Gen}}^{\text{SR}},\\ L_{\text{MSE}}^{\text{SR}}=\frac{1}{r^{2}WH}\sum_{i=1}^{rW}\sum_{j=1}^{rH}{\left(Y_{i,j}-G_{\theta_{G}}{\left(X\right)}_{i,j}\right)}^{2},\\ L_{\text{Gen}}^{\text{SR}}=\sum_{n=1}^{N}-\log D_{\theta_{D}}\left(G_{\theta_{G}}\left(X\right)\right), \end{array}$$

where *a*_1_ and *a*_2_ are weighting parameters; *L*_MSE_^SR^ denotes the content loss which is the most widely used optimization target for image SR on which many state-of-the-art approaches rely; *L*_Gen_^SR^ denotes the adversarial loss of generative network, which tries to fool the discriminator network; *r* is the downsampling factor in the downsampling operation; and *W* and *H* denote the size of the image, respectively.

## 4. Experiments

In this section, experiments are performed to qualitatively and quantitatively evaluate the proposed method. Also, the quantitative evaluation is based on PSNR and SSIM [39] in this work.

### 4.1. Medical Image Datasets

In this work, a database suitable for medical image SR is constructed by integrating the following medical images: the Brain, Lung, Abdomen, and Bone. 250 images for each of these four body parts are used in the database. Brain and Lung images are chosen from the Cancer Imaging Archive (TCIA) [40]. Bone and Abdomen images are provided by the radiology department of a hospital in China. The training set is composed of 175 images for each part, i.e., 700 medical images in total; the test set is made from the remaining 300 images.

In addition, we select two publicly available COVID-19 CT datasets, termed as COVID-CT_349 (https://github.com/UCSD-AI4H/COVID-CT) including 349 images and COVID-CT_19 (https://github.com/ieee8023/covid-chestxray-dataset) including 19 images. We use COVID-CT_349 as the training set and COVID-CT_349 and COVID-CT_19 as the test sets, respectively.

### 4.2. Implementation Details

For the constructed medical image database, the 700-image training dataset is used for the data augmentation. Following [4, 5], the original training images are first rotated by 90°, 180°, and 270° and then flipped horizontally. Therefore, we have seven additional augmented versions for each original image. The same data augmentation method is performed on COVID-CT_349 and COVID-CT_19.

We run the experiments on HP 7920 series tower server with NVIDIA RTX3090 graphics card. We use Adam optimizer to train the proposed model. The initial learning rate is set to 0.0001 for all layers and decreased by half after every 50 epochs. The proposed model converges after 200 epochs. The training procedure takes roughly 9 hours on a single Tesla P40 GPU.

### 4.3. Comparison with State-of-the-Art Methods

In this section, the performance of the proposed method is evaluated on both the constructed medical image database (i.e., Brain, Lung, Abdomen, and Bone) and COVID-19 datasets. For a straightforward test, the published codes of other models and the same training set are used for all methods. Tables 1–6 show the comparison results of PSNR and SSIM values for scales 4 and 8, indicating that the proposed Med-SRNet obtains higher PSNR and SSIM values on these datasets on average compared with other methods. Bold indicates the best.

**Figure 4:**
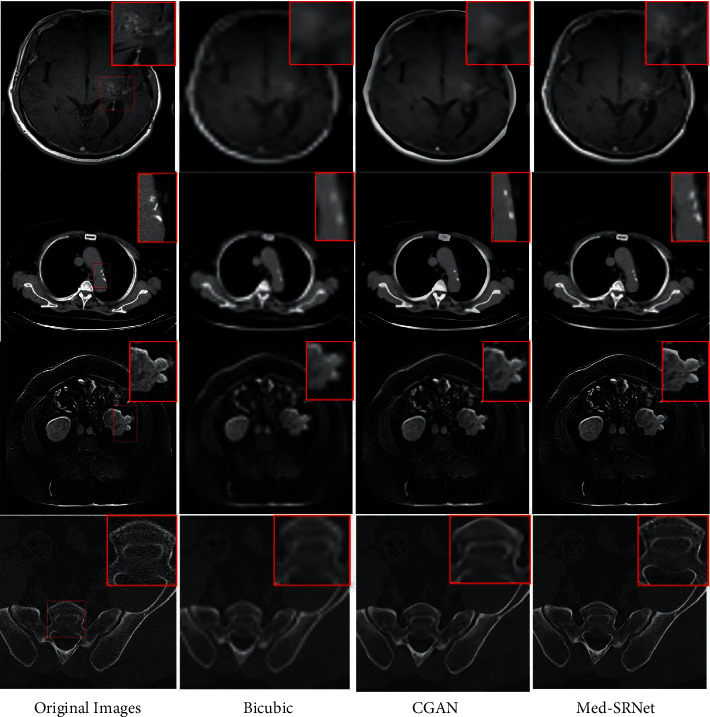
Qualitative results on the constructed medical image database. 1–4 columns are the original images, bicubic interpolation (8×) images, SR images by CGAN, and SR images by the proposed Med-SRNet.

**Figure 5:**
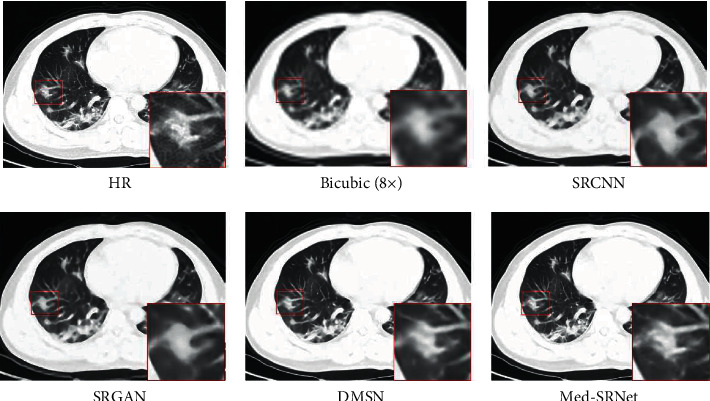
Qualitative results on a COVID-19 image for different methods.

Figure 4 presents patterns of scale 8 for four image datasets, i.e., Brain with suspected cerebrovascular malformation, Lung with atherosclerosis of aorta of pulmonary mediastinal window, Abdomen with renal cyst, and normal Bone sites. The images reconstructed by the proposed Med-SRNet have a clearer structure and abundant detail, which is obviously visible in the zoomed regions.  Figure 5 shows the patterns of scale 8 for COVID-19 images with the characterization of ground-glass opacities. It is easy to find that the proposed Med-SRNet obtains better results than other methods in detail recovery.

### 4.4. Ablation Study

This section evaluates the performance of feature aggregation component on the constructed medical image database. Compared to SRGAN [18] and HRNet [30], the proposed feature aggregation part adds one component: deconvolution. The comparison (scale: 8×) of PSNR for different feature aggregation parts is shown in Table 7. Our method obtains higher PSNR on average. “BI” and “MR” are the abbreviations of upsampled bilinear interpolation operation and multi-resolution, respectively.

## 5. Conclusion

We present a GAN-based medical image SR network via HR representation learning. It effectively exploits features of medical images to boost the SR performance considering the advantages of GAN that can significantly reconstruct the visual quality of the images. It is important that HRNet is employed as backbone of SR to maintain the HR representations and repeat multi-scale fusions to strengthen HR representations for facilitating SR. Also, deconvolution operations are adopted to recover HR representations from the LR images with the aim to yield richer aggregated features, instead of simple bilinear interpolation operations used in HRNetV2. Experimental results qualitatively and quantitatively illuminate that the proposed method is superior to other leading edge ones in LR image restorations. In the future, we will study superior multi-scale transform methods, which integrate SR task to better exploit features from medical images.

## Figures and Tables

**Table 1:** PSNRs for various methods on the constructed database.

Datasets	Scale	Bicubic	DWSR [32]	IDN [14]	MSRN [15]	SRFBN [16]	DMSN [25]	Med-SRNet
Brain	4	26.517	28.268	28.454	28.841	28.864	28.964	**29.948**
	8	22.338	23.926	24.184	24.317	24.586	24.733	**25.385**

Lung	4	27.338	29.196	29.323	29.455	29.538	29.958	**31.164**
	8	23.795	25.027	25.231	25.402	25.545	25.685	**26.335**

Abdomen	4	27.902	29.934	30.341	30.407	30.581	30.523	**31.106**
	8	24.694	26.095	26.199	26.325	26.564	26.591	**27.515**

Bone	4	26.414	28.451	28.555	28.555	28.642	28.685	**29.494**
	8	24.478	25.759	25.231	25.790	25.972	26.031	**26.536**

**Table 2:** PSNRs for various methods on the constructed database.

Datasets	Scale	Bicubic	SRGAN [18]	DCGAN [20]	CGAN [23]	Med-SRNet
Brain	4	26.517	28.154	28.685	29.374	**29.948**
	8	22.338	23.635	24.361	24.952	**25.385**

Lung	4	27.338	29.162	29.548	30.356	**31.164**
	8	23.795	25.014	25.432	25.744	**26.335**

Abdomen	4	27.902	29.634	30.597	30.673	**31.106**
	8	24.694	25.968	26.485	26.900	**27.515**

Bone	4	26.414	28.361	28.763	28.822	**29.494**
	8	24.478	25.657	25.528	26.165	**26.536**

**Table 3:** SSIMs for various methods on the constructed database.

Datasets	Scale	Bicubic	DWSR [32]	IDN [14]	MSRN [15]	SRFBN [16]	DMSN [25]	Med-SRNet
Brain	4	0.831	0.865	0.868	0.872	0.874	0.875	**0.892**
	8	0.704	0.755	0.759	0.763	0.755	0.764	**0.799**

Lung	4	0.825	0.869	0.871	0.874	0.874	0.879	**0.891**
	8	0.739	0.779	0.783	0.786	0.788	0.791	**0.811**

Abdomen	4	0.796	0.852	0.856	0.857	0.865	0.865	**0.872**
	8	0.673	0.717	0.722	0.728	0.731	0.730	**0.748**

Bone	4	0.427	0.644	0.649	0.649	0.652	0.661	**0.673**
	8	0.342	0.368	0.783	0.372	0.375	0.380	**0.386**

**Table 4:** SSIMs for various methods on the constructed database.

Datasets	Scale	Bicubic	SRGAN [18]	DCGAN [20]	CGAN [23]	Med-SRNet
Brain	4	0.831	0.858	0.862	0.880	**0.892**
	8	0.704	0.741	0.753	0.776	**0.799**

Lung	4	0.825	0.861	0.869	0.881	**0.891**
	8	0.739	0.763	0.778	0.795	**0.811**

Abdomen	4	0.796	0.846	0.852	0.860	**0.872**
	8	0.673	0.705	0.719	0.740	**0.748**

Bone	4	0.427	0.624	0.640	0.666	**0.673**
	8	0.342	0.361	0.775	0.381	**0.386**

**Table 5:** PSNRs for various methods on COVID-19 datasets.

Datasets	Scale	Bicubic	SRCNN [3]	SRGAN [18]	DMSN [25]	Med-SRNet
COVID-CT_349	×4	26.573	30.025	32.242	32.716	**34.083**
COVID-CT_349	×8	24.047	26.219	27.884	28.026	**28.868**
COVID-CT_19	×4	30.963	33.795	35.692	35.714	**36.643**
COVID-CT_19	×8	27.125	28.246	29.437	29.503	**29.901**

**Table 6:** SSIMs for different methods on COVID-19 datasets.

Datasets	Scale	Bicubic	SRCNN [3]	SRGAN [18]	DMSN [25]	Med-SRNet
COVID-CT_349	×4	0.681	0.732	0.771	0.783	**0.804**
COVID-CT_349	×8	0.627	0.679	0.708	0.716	**0.753**
COVID-CT_19	×4	0.832	0.868	0.906	0.925	**0.942**
COVID-CT_19	×8	0.764	0.802	0.840	0.857	**0.881**

**Table 7:** Comparison of PSNR for different feature aggregations.

Methods	Datasets			
	Brain	Lung	Abdomen	Bone
Baseline1 [18]	23.926	25.014	25.968	25.657
Baseline2 [30]	24.956	25.751	26.982	26.128
Baseline2 + BI MR fusion [31]	24.993	25.814	27.106	26.196
Baseline2 + deconvolutions	25.025	25.957	27.198	26.243
Baseline1 + baseline2 + deconvolutions (ours)	**25.385**	**26.335**	**27.515**	**26.536**

## Data Availability

The data used to support the findings of this study are included within the article.
